# Knowledge, Attitudes, Intentions and Vaccine Hesitancy among Postpartum Mothers in a Region from the Northwest of Romania

**DOI:** 10.3390/vaccines11121736

**Published:** 2023-11-21

**Authors:** Camelia Florina Iova, Dana Badau, Mădălina Diana Daina, Corina Lacramioara Șuteu, Lucia Georgeta Daina

**Affiliations:** 1 Faculty of Medicine and Pharmacy, Doctoral School, University of Oradea, 1 December Sq., 410081 Oradea, Romaniadiana_daina98@yahoo.com (M.D.D.); 2 Faculty of Physical Education and Mountain Sports, Transilvania University, 500068 Brasov, Romania; 3 Department of Psycho-Neurosciences and Recovery, Faculty of Medicine and Pharmacy, University of Oradea, 1 December Sq., 410081 Oradea, Romanialucidaina@gmail.com (L.G.D.)

**Keywords:** vaccination knowledge, vaccine hesitancy, positive attitudes, hesitant behavior

## Abstract

This study aims to identify the presence of vaccine hesitancy and the factors that could have determined it in a group of mothers in the postpartum period, with an evaluation of both the level of knowledge and information, as well as the attitudes, perceptions, intentions and sources of information about vaccination. The study was based on a survey—Vaccine Hesitancy Identification Survey—applied in two maternity wards from Bihor County and structured into six subscales (34 items). Based on the answers to the key questions (“*Which of the following statements best describes your plans for vaccinating your child?*”—item 1 of subscale 4; “*Overall, how hesitant do you consider yourself to be about vaccinating your child?*”—item 4 of subscale 4), we identified two groups: the group of mothers without hesitant behavior (non-hesitant), called the group pro vaccine (GPV), and the group of mothers with hesitant behavior, called the group non vaccine (GNV). Vaccine hesitancy was identified in our study in 47.28% of the participants (191 of the 404 mothers included). Most of them come from an urban environment (57.59%), have university and post-secondary education (58.64%) and are prim parous (58.64%). The behavior of participants from GNV is influenced by a low level of knowledge and information regarding vaccination and by concerns related to adverse reactions, new vaccines and the number of vaccines administered. Also, this group is characterized by an increased perception of the risks related to vaccination, while the perception of the risks associated with the disease is low. For all subscales, important differences were registered between the two groups in favor of GPV, a group characterized by positive attitudes and perceptions and a better level of knowledge compared to GNV. This study aims to represent a starting point for the organization and running of information campaigns regarding vaccination at the level of Bihor County, especially in areas with low vaccination coverage, where this behavior is identified.

## 1. Introduction

Vaccination is and will remain one of the most important public health interventions worldwide, with immunization programs proving successful over time in eliminating and eradicating severe, potentially fatal diseases [1]. However, despite the proven success of immunization programs globally, we are faced with an increasingly negative attitude and skepticism towards vaccines and vaccination. Refusing or delaying vaccination has, over time, led to a re-emergence of vaccine-preventable diseases such as measles or whooping cough; diseases that were once almost eliminated [2]. There are and will continue to be people who will doubt the importance and necessity of vaccines, and express concerns about their safety. Moreover, studies show a decrease in the population’s confidence in vaccines, a fact that has become increasingly evident in the context created by the COVID pandemic. Thus, vaccination has become a victim of its own success: the benefits are clear, without room for interpretation, but, paradoxically, the number of those who consider vaccines useless, even dangerous, is increasing; by decreasing incidence, eliminating or even eradicating some diseases, the false perception was created that the risks are very small or non-existent or that vaccination is not important or necessary [3,4,5,6].

The SAGE Working Group on Vaccine Hesitancy defined “vaccine hesitancy” (VH) as a “delay in acceptance or refusal of vaccination despite availability of vaccination services”. The term “vaccine hesitancy” can be translated as “hesitant behavior” and generally refers to situations in which individuals have doubts and concerns about vaccination. The definition is much wider than the official one proposed by experts and VH can be interpreted as a state of uncertainty, anxiety, or concern that occurs when an individual has to make a decision about vaccination. As Larson et al. point out in a recently published article, VH is “an attitude or feeling”, while vaccination is an “action” that is then objectified in vaccine coverage [5,6,7,8,9]. There is no single reason that can cause doubts, concerns, or worries about vaccines and vaccination. VH is present in the community, regardless of the type of population, ethnicity or geographical boundaries, and regardless of the type of vaccine, route of administration, number of doses or possible adverse reactions. The determining factors of this behavior are multiple. The World Health Organization requested member states, through the United Nations Children’s Fund Joint Reporting Form (JRF), for a report on the impact of vaccine hesitancy in these countries; of the 194 countries that completed the form, 74% reported that this phenomenon is a major cause for concern, affecting vaccination rates and increasing the risk of outbreaks of vaccine-preventable diseases [10]. An analysis carried out over a three-year period revealed that the top three reasons for VH worldwide were risk–benefit ratio (safety concerns, fear of side effects), lack of knowledge about vaccination and awareness of the importance of vaccination, as well as issues related to religion, culture, gender, and socioeconomic level [11]. The current state of confidence in vaccines and vaccination in the European region (EU-27), evaluated on a sample of 25,143 people from the general population and 3012 medical professionals, highlights the fact that in the year 2022, in the general population, 81.5% and 85.6% of respondents believe/agree that vaccines are important and effective, respectively, which shows a decrease from 91.8% and 89.7%, respectively, in 2020 and 89.6% and 87.2%, respectively, in 2018. The percentage of participants who consider vaccines safe was 82.3% in 2022, which represents an increase from 81.9% in 2018, but a decrease compared to 2020 (87%).

Romania has also seen a decline in the percentage of people who believe vaccines are important (from 87.8% in 2018 to 84.4% in 2022), safe (from 81.9% in 2018 to 77.7% in 2022) or effective (from 84.7% in 2018 to 82% in 2022) [12]. Moreover, the data reported by UNICEF show a decrease in the confidence of the Romanian population in childhood vaccines of up to 10% during the COVID pandemic [13]. A survey carried out at national level in 2019 on a sample of 2115 people highlighted the fact that 81.1% of respondents considered vaccination of children necessary and more than half of them (54.9%) were of the opinion that the benefits of vaccination outweigh the potential risks; 54.1% of the participants were aware that vaccine-preventable diseases are serious, but less than half (49.1%) considered vaccination to be the most effective and safe method of protection against these diseases; also, 28.7% believed that “ vaccination is a complicated issue and must be evaluated on a case-by-case basis”. Only 39.7% of those surveyed indicated that they trust the information they receive about vaccines. 62.7% of respondents believed that vaccination should be mandatory for access to collectivities [14]. Combating VH is a difficult task because of the multiple factors that can influence an individual in the process of making a decision to vaccinate themselves or their child. Unfortunately, regaining public confidence in vaccination is not easy and there is no single winning strategy; a multi-pronged approach is required. Regardless of the difficulties, the risks to public health are too great and immediate action is needed [15,16].

The present study is a prospective analytical observational one, the main purpose being to evaluate knowledge about vaccination, as well as the attitude of parents in the process of making a decision regarding the immunization of their own children, with an emphasis on the perception of the safety, effectiveness and importance of vaccines and vaccination. The study evaluates not only knowledge and attitudes but aims to identify the factors that determine hesitant behavior among parents, behavior that is reflected in the refusal or postponement/delay of vaccination. Through the principal components analysis technique, we identified patterns that could explain to a certain extent the behavior of the mothers in the two groups of the study. The project and methodology of this study were developed before the onset of the pandemic and followed the measles epidemic that evolved in the period 2016–2019 and resulted in over 20,000 cases in Romania, of which 64 led to deaths, in the context of a vaccination coverage far below the level of 95% necessary for protection by ensuring herd immunity; thus, vaccination coverage with two doses of measles, mumps and rubella vaccine (MMR) was, in August 2016, at a percentage of 76.3% at the age of 5 years and 72.6% at the age of 7 years; low vaccination rates were also recorded for the other types of vaccine, except for bacillus Calmette-Guerin (BCG) vaccine. Vaccination rates accentuated their decline during the pandemic years and will continue to decline in the absence of prompt and specific strategies [17,18,19]. 

In Romania, information about vaccination is provided to the general population by healthcare professionals of different specialties, especially family physicians. In 2023, the Ministry of Health launched a nationwide informational and awareness campaign about the importance of vaccination (five audio and video materials), targeting the general public, under the slogan “I do not only hope, I know I will be ok! I am getting vaccinated!” [20]. 

The study can be the starting point for a series of public health interventions at the county level and beyond that are vital in order to increase the population’s confidence in vaccines and vaccination, to respond to doubts, concerns, worries and hesitancy regarding vaccination and to bring vaccination coverage rates to levels that ensure community-wide protection.

## 2. Material and Methods

### 2.1. Study Design

The study is part of broader research on attitudes and perceptions related to vaccination, representative of the doctoral thesis of the first author, which took place in several stages and was completed in July 2023. The present study was conducted on a random and representative sample of mothers hospitalized after giving birth, and it took place in two hospitals in Bihor County (one public hospital and one private). We chose this moment because, in the postpartum period, mothers have to make the first decisions regarding the vaccination of their children; also, studies show that this may be the optimal time to address potential concerns about vaccination and to provide mothers with information to help them take a decision regarding the administration of the first vaccines and those who will later complete the vaccination schedule of the child, according to the National Vaccination Program.

In the first stage, we obtained agreement from the two hospitals to conduct the study and apply the questionnaires. In the second stage of the research, mothers’ eligibility was checked daily; those that met the inclusion criteria and completed the informed consent form were included in the study. Most of the questionnaires in print format were distributed and collected by the principal investigator or, in certain situations, by a designated person within the health unit trained in this regard. Before and after collecting the questionnaires, the principal investigator was available for clarification regarding the questions, and, at the end, discussions with mothers who wanted to know more about vaccines and vaccination. For each participant, we randomly assigned an identification code, kept and used in all stages of the study. In the next step, all the data were entered into an Excel file (Excel Office 2019), respecting the previously established coding. In the fourth stage, the two groups were identified, based on the predetermined methodology.

So, considering the answers to the key questions (“Which of the following statements best describes your plans for vaccinating your child?”—item 1 of subscale 4; “Overall, how hesitant do you consider yourself to be about vaccinating your child?”—item 4 of subscale 4), we identified, in accordance with the study plan after applying the questionnaire, two groups: the group of mothers without hesitant behavior (those who answered “I intend to vaccinate my child with all the vaccines recommended by the doctor AND I don’t hesitate at all”), called the group pro vaccine (GPV), and the group of mothers with hesitant behavior (those who answered “I plan to vaccinate my child with some vaccines, but not all” or “I do not plan to vaccinate my child” or “I haven’t decided yet/I don’t know” to the first question, and “not very hesitant” or “not sure” or “somewhat hesitant” or “very hesitant” to the second key question), called the group non vaccine (GNV).

### 2.2. Participants

The final group was represented by 404 postpartum mothers and their children (413). Inclusion criteria: mothers hospitalized after childbirth, aged 18 years or older, who gave written consent to answer the questions in the questionnaire by filling in the informed consent form; mothers who answered all the questions and gave birth to healthy children; mothers living in Bihor County. Exclusion criteria: age under 18, residence in another county.

### 2.3. Instrument of Evaluation

The questionnaire for this research was developed by a team that involved the authors and other medical professionals, after consulting and reviewing the specialized literature [21,22,23,24] and it was based on pre-existing questionnaires. The questionnaire was pretested on a sample of 20 mothers from the public maternity ward. The responses from this sample were not included in the study analysis. 

Exploratory factor analysis was performed on these 20 questionnaire responses. The cumulative proportion of variance explained by the six factors was 0.76 and the sum of squared loadings for each factor was greater than 1 (Table 1). Only items with a factor loading greater than 0.5 were retained in the final form of the questionnaire.

The applied questionnaire assesses interest and familiarity with the main types of vaccines and with the national immunization schedule, past and present attitudes regarding immunization and vaccine-preventable diseases, intention to vaccinate or not vaccinate the child, reasons for refusing or postponing vaccination, confidence in the efficacy, safety and the benefits of vaccination, worries and concerns that may lead to hesitancy, sources of vaccination information. 

The questionnaire was structured in two parts—one general and one specific. The general part was in turn divided into two categories: demographic data and general characteristics (mother’s level of education, occupation, age, background, marital status, number of children—prim parous or multiparous), as well as previous experiences related to vaccination (refusing to vaccinate one of their children in the past, motivation). The second specific part consisted of a survey that we renamed the Vaccine Hesitancy Identification Survey, with 34 items, organized into six subscales: subscale 1—knowledge about vaccination, general perceptions and perception of disease severity (six items), subscale 2—perception of the importance, effectiveness, safety and benefits of vaccination (seven items), subscale 3—worries, concerns, perception of vaccination risk and disease risk (six items), subscale 4—vaccination intentions (four items), subscale 5—trust in medical professionals (three items), subscale 6—sources of information regarding vaccination (eight items). The study and the questionnaire were approved by the Ethics Committee of the University of Medicine and Pharmacy “Iuliu Hațieganu” Cluj Napoca—approval no. 412/14.11.2018 and by the Ethics Committee of Oradea County Emergency Clinical Hospital—approval no. 1534 of 17.01.2019.

### 2.4. Statistical Analysis

The statistical processing of the data was carried out using SPSS–IBM 24. The answers to the questionnaire items were coded according to the number of answer options; study data were presented numerically and as a percentage. Principal component analysis (PCA) is a variable reduction technique that has many similarities to exploratory factor analysis. Its purpose is to reduce a larger set of variables into a smaller set of “artificial” variables, called “principal components”, that account for most of the variation in the original variables; we used this technique to identify certain behavioral typologies of the groups (behavior patterns). The identified behavioral patterns were made for each group and subscale of the study questionnaire. Only items that had positive values higher than 0.7 for a factor were considered to have directly contributed to shaping the behavioral pattern. An initial analysis was run to obtain Initial Eigenvalues for each component of each subscale of the questionnaire, respectively, for each group included in the study. The summed percentage values of the components for each group of subjects explain the percentage value of the variance from the total percentage of the group (100%).

## 3. Results

### 3.1. Personal Data of the Sample

Of the 404 mothers included in the study, 213 (52.72%) were classified as having non-hesitant behavior (GPV), while 191 (47.28%) were considered as hesitant (GNV) (Table 2). No important differences were identified between the two groups in terms of mean age (28.99 versus 29.43). Regarding area of origin, rural mothers predominate in GPV compared to GNV when reporting the total sample (57.59% versus 42.41%). Most mothers who graduated from general school (71.05% when reporting the total sample), as well as those without studies, are part of GPV. Of the 19 Roma mothers, 15 (78.95%) were included in GPV. The majority of mothers who have occupations in the medical field are part of GNV (61.76% versus 38.23%). Housewives predominate in GPV (58.65% compared to 41.35%). Also, the majority of first-time mothers belong to GNV (53.85% versus 46.15%).

### 3.2. Subscale 1—Knowledge about Vaccination, General Perceptions and Perception of Disease Severity

A better level of knowledge is highlighted among GPV participants compared to GNV. Thus, 85.91% and 89.20% of the mothers in the first group heard about the fact that vaccines prevent serious diseases and believe that this statement is true, respectively, compared to 79.06% and 72.77% of the participants in GNV, respectively. Furthermore, 95.77% of GPV mothers compared to 82.72% of GNV participants believe that vaccines can protect their own child against severe diseases. Also, 31.45% of mothers in the non-hesitant group had heard about all vaccines in the program, compared to 21.46% in the hesitant group. Regarding mandatory vaccination, 87.79% versus 50.78% of the participants from the two groups, in favor of GPV, believe that vaccination should be mandatory. The results of the statistical processing of the main components (PCA) highlight two behavioral patterns for the two groups. We have named each component according to the best result, thus identifying which is the item that influences the behavior the most. Thus, for GNV, the first behavioral pattern was outlined in the answers to *S1.2. Vaccines prevent serious, potentially fatal diseases* with 0.787 points, and the second in the answers to *S1.4. How familiar are you with/know your child’s current recommended vaccination schedule?* with 0.672 points. The GPV sample also registered two components, defined in *S1.2. Vaccines prevent serious, potentially fatal diseases* with 0.733 points and *S1.6. Do you think vaccination of children should be compulsory?* with 0.784 points (Table 3). The two components of GNV explain 52.8% of the variance; the two components of GPV combined explain 50.8% of the variance. 

### 3.3. Subscale 2—Perception of the Importance, Effectiveness, Safety and Benefits of Vaccination

For all subscale 2 items, positive responses predominate in GPV when compared to GNV. Thus, 99.06% compared to 78.54% consider it very important/important for the child to receive all the vaccines offered by the program, and 97.65% compared to 83.77% believe that vaccines are very important/important, effective (94.83% versus 81.15%), safe (83.10% versus 50.26%.) and beneficial (90.14% versus 64.40%). Also, the percentages of mothers who answered “*I do not know*” to the subscale items are higher in GNV, highlighting a low level of information and low interest in vaccines and vaccination. For subscale 2, one behavioral pattern was identified for each sample; thus, for GNV, the answers to S2.7. *Vaccination is a very good way to protect my child* explain the behavior of the participants in this group in a proportion of 70.52%, while for GPV the pattern is defined by the answers to S2.6. *All vaccines given to my child/community through the vaccination program are beneficial* in a proportion of 57.06% (Table 4). The single component of GNV explains 70.5% of the variance; the component of GPV explains 57% of the variance. 

### 3.4. Subscale 3—Worries, Concerns, Perception of Vaccination Risk and Disease Risk

Mothers in the group characterized by hesitant behavior (GNV) are more concerned when it comes to post-vaccination adverse reactions or the safety of new vaccines. Also, the perception of disease risk is lower among mothers in GNV; thus, only 19.89% (GNV) compared to 42.25% (GPV) believe that their child needs to be vaccinated even if the disease is considered to be rare. A considerable number of participants answered “*I do not know*”, especially in the group with hesitant behavior, which again indicates a lack of interest, but also of information. Two behavioral typologies were highlighted for GNV, reflected in the answers to S3.4. *Children receive more vaccines than they should* and S3.2. *I am concerned about side effects*, with percentages of variance of 40.74% and 17.48%, respectively, and a behavioral pattern for GPV, also reflected in the answers to S3.4. *Children receive more vaccines than they should*, with percentages of variance of 40.61%. The two combined components of GNV explain 58.2% of the variance; the single component of GPV explain 40.6% of the variance (Table 5). 

### 3.5. Subscale 4—Vaccination Intentions

As we can see from the percentage distribution of responses in the two studied groups, vaccination intentions in the group of mothers without hesitant behavior (GPV) are positive, whether it is about vaccines in general or about vaccines newly introduced in the National Vaccination Program (anti-pneumococcal). For this subscale, the same behavioral patterns were identified in both GPV and GNV; thus, the behavior of the two groups is mainly reflected in the answers to items S4.2. *I plan to vaccinate my child with the pneumococcal vaccine, a new vaccine introduced in the vaccination schedule, to protect against pneumococcal pneumonia* and S4.3. *Would you like to vaccinate your child with other types of vaccine, which are not included in the national program?*; GPV behavior shows mothers’ willingness to vaccinate their child with pneumococcal vaccine and other optional vaccines in greater numbers compared to GNV (Table 6). The two combined components of GNV explain 64.2% of the variance; the single component of GPV explains 54% of the variance. 

### 3.6. Subscale 5—Trust in Medical Professionals

Confidence in the information provided about vaccines, in the doctor’s recommendation and willingness to talk to the doctor when concerns and questions about vaccines and vaccination arise are present in both groups, but in different proportions. Thus, GPV recorded higher values of affirmative answers for all items. The behavior of the participants in both groups is influenced (66.20% and 63.52% respectively) as shown in the answers to S5.2. *I generally do what my doctor recommends when it comes to vaccinating my children*, as indicated by the principal components analysis; thus, mothers in both samples make vaccination decisions based on physician recommendation (Table 7). The component of GNV explains 66.2% of the variance; the single component of GPV explains 63.5% of the variance. 

### 3.7. Subscale 6—Sources of Information

Regarding the sources of information, the family doctor is indicated as a source of information by 33.80% of the participants in GPV and 22.51% of the mothers belonging to GNV, respectively. The majority of respondents from GPV (94.84%), but also from GNV (90.58%), say that they were not influenced in making a decision by certain information they saw or read, and 84.51% of respondents from GPV, compared to 61.25% from GNV, believe that they have enough information or sources available to make a correct decision. Mothers would like to receive more information about vaccines and the diseases they prevent, regardless of the group they belong to. The doctor is indicated as the most reliable source of information by most mothers participating in this study (97.65% in GPV and 95.29% in GNV). The behavior of the participants is reflected, both for GNV and for GPV, in a proportion of 20.20% and 21.30%, respectively, in the answers to S6.2. *Where did you look/who did you talk to?.* Another behavioral component for GNV is explained for 18.28% of mothers in the answers to S6.4. *Did certain information you heard/seen make you change your mind about the decision to vaccinate your child?*, and for GPV in the answers to S6.5. *If a public figure, especially one you look up to, advocated against vaccination, would that influence your decision to vaccinate your child?* (Table 8). The two combined components of GNV explain 38.4% of the variance; the two components of GPV combined explain 38.3% of the variance. 

## 4. Discussions

In this study, we tried to identify the presence of vaccine hesitancy in a sample of mothers in a beautiful but delicate period, which was new to some of them: the moment of the birth of a child, a moment which, in addition to the inherent joy and emotion, comes with a series of responsibilities and the need to make important decisions. Among them, one of the most important—if not the most important—is related to the health of one’s own child and implicitly to his vaccination. The final goal was to understand the reasons behind vaccine hesitancy and what differentiates the group of mothers with a hesitant behavior from mothers who have positive attitudes and perceptions regarding vaccination, in order to be able to effectively intervene in this population segment. Hesitant behavior was identified in our study in 47.28% of the participants. Most of them come from the urban environment (57.59%), have university and post-secondary education (58.64%), are married (91.10%) and have different occupations (22.51% are housewives, 10.99% work in the medical field—medical personnel/pharmacists) and 58.64% had experienced their first birth (prim parous).

The group of mothers with hesitant behavior (GNV) includes those participants who indicated, through the answers given to the questions of the Vaccine Hesitancy Identification Survey, that they did not decide/don’t know/do not intend to vaccinate their child or only intend to do so with certain types of vaccine, and those who declared themselves very hesitant/somewhat hesitant/not very hesitant or unsure about their intention to vaccinate. On the other hand, the group without hesitant behavior (GPV) consists of those mothers who indicated that they intend to vaccinate their child with all the recommended vaccines and did not hesitate at all in making this decision. In the first stage, by structuring specific subscales, the level of knowledge and perceptions regarding vaccines and vaccination in the two groups were tracked, especially those regarding the importance, effectiveness, safety and benefits of vaccination, but also the perception of risks related to the disease, respectively vaccination; vaccination intentions, trust in health professionals, as well as the main sources of information about vaccination were assessed. The data show a higher level of knowledge in GPV compared to GNV, with mothers in GPV having better knowledge about vaccines in the national calendar and being more aware of the role of vaccines in preventing severe, potentially fatal diseases. However, compared to the total sample, we found that over 25% of the mothers did not know that there is a vaccination schedule or what the vaccines in the calendar are, and 47.28% had relative information (they heard about a few). The high percentage of those who did not have accurate knowledge about the vaccination schedules reflects a low level of information possessed by the population included in the study, the deficiencies being greater in the group with hesitant behavior (GNV). Also, although the majority of mothers from both groups (84.51% in GPV and 61.25% in GNV) claim that they have sufficient information or sources of information about vaccination, the answers to the questions do not confirm these statements, especially for mothers from GNV. The high percentage of mothers who answered “*I don’t know*” to the items of subscales 1–3, especially in GNV, again indicates a low level of knowledge and information regarding vaccination.

If we approach the topic of mandatory vaccination, there are also important differences between the groups, with GPV mothers leaning in favor of it. Positive perceptions and attitudes regarding the importance, effectiveness, safety and benefits of vaccines prevail in GPV compared to GNV. Analyzing the distribution of responses and percentages for the two samples, it is highlighted that the participants from GNV are the most concerned about potential adverse reactions, new vaccines or the number of vaccines administered and have a higher perception of the risk related to vaccination compared to GPV, while the perception of disease risk is lower. Mothers who are part of GPV demonstrate a higher level of trust in the information received and in the doctor’s recommendation regarding vaccination and are more open to discussing concerns and worries related to vaccination. Most of the participants, regardless of the group, searched for information about vaccines and vaccination at some point. The participants from the pro-vaccine group prefer to turn to the family doctor (33.80%), while the participants from GNV report in the highest proportion multiple sources of information (41.36%). The analysis of the principal components highlights, based on the answers to the survey questions, certain patterns that could explain the behavior of the participants from the two groups.

Thus, the behavior of participants in GNV is influenced by the low level of knowledge regarding vaccines and vaccination (most have heard about some of the vaccines in the calendar or did not know that there is a vaccination calendar or which are the recommended vaccines) and by concerns regarding the adverse effects of vaccines; most do not know if children receive more vaccines than necessary, but some are convinced that they do. Most of the mothers in GNV are undecided about vaccination with vaccines newly introduced in the calendar (anti-pneumococcal) or with optional vaccines. What can tip the balance in a positive direction, in favor of vaccination when the intention expressed in the answers to the questionnaire items will turn into a decision, is the fact that most of these mothers believe that vaccines can still prevent serious diseases and that vaccination could be a good method to protect their child in the future. Also, behavior can be decisively influenced by the doctor’s recommendation, with mothers from GNV indicating that, in general, they follow his advice.

Also, what is interesting is the fact that the majority of mothers from GNV who have heard and believe vaccines prevent serious illnesses, as well as most of the participants in this group worried about the side effects, come from urban areas. We can comprehend from these findings that the concerns regarding potential adverse reactions outweigh their understanding of the vaccines’ role in preventing diseases, which might explain their hesitation.

The behavior of GPV mothers is also reflected in different proportions in the answers to certain questions and can be explained by the belief that vaccines prevent serious diseases, that all vaccines recommended and offered through the program are beneficial and by the fact that mothers in this group follow the doctor’s instructions related to vaccination. Most mothers in GPV believe that vaccination should be mandatory and intend to vaccinate their child with new vaccines, such as the pneumococcal one.

The study is limited as a representative sample for a certain region; a study at a regional or national level, which not only evaluates attitudes, but which proposes specific interventions, followed by their implementation at the population level, is imperative to respond to the hesitating behavior and the increasingly accentuated lack of trust in vaccines, medical personnel, authorities, decision-makers and mistrust augmented by the pandemic and reflected in ever-lower vaccination rates at the national level.

Also, in our research, we focused on mother’s perceptions and attitudes regarding vaccination, but when it comes to a decision about the child’s vaccination, fathers’ involvement is important and also adolescents’ regarding the HPV vaccination.

A series of studies from the specialized literature address the topic of parents’ hesitant behavior in the process of making a decision regarding vaccination. In general, these studies evaluate the presence of hesitant behavior in a certain representative sample; in the present study, we compared two distinct groups in terms of attitudes related to vaccination (a non-hesitant group, GPV, and a hesitant group, GNV). Our study also highlights certain behavioral typologies that could explain these attitudes. 

Dube et al. [25] and Seskute et al. [26] also conducted studies that included mothers in the postpartum period. Fifteen percent of the mothers from the first research presented an elevated VH score, while one third of the participants had an intermediate score [25]. Napolitano et al. identified the presence of VH in 34.7% of the participants in a group of parents of children aged between 2 and 6 years. Young mothers with a lower level of education, who believe that most vaccine-preventable diseases are not severe, are concerned about possible side effects or the safety of vaccines, have delayed or refused at least one vaccine, or do not trust in pediatricians are those who have been identified as having a hesitant attitude [27]. 

Melot et al. conducted a study to assess the presence of VH in a province in Italy; in this study, 18.7% of participants were defined as hesitant; among those who showed hesitant behavior, 33% declared that they did not have enough information, and 11.3% declared themselves completely against vaccination [28].

Another study that assessed the factors associated with VH among parents reported a prevalence of VH of 13.8%; in line with our research, the authors reported a significant relationship between the level of education and hesitant behavior. Thus, mothers with a university education were the ones who expressed hesitancy about vaccinating their children. VH was also more common in parents who reported getting their vaccination information from the Internet or the media [29]. In another study [30], 11% of parents were identified as very hesitant/hesitant about vaccination.

Kempe et al. reported a prevalence of VH regarding routine childhood vaccinations of 6.1% [31]. Only 3% of mothers included in a study conducted in Cyprus showed hesitant behavior regarding vaccination [32].

Hadjipanayis et al. reported in a study that assessed parents’ confidence in vaccines in 18 European countries a percentage of parents who did not hesitate at all of 56%, while 24% declared themselves somewhat hesitant and 4% very hesitant regarding vaccination. Unlike other studies, including ours, vaccine hesitancy scores were higher for parents with elementary or high school education, compared to those with university education. [33].

As in our study, most participants in other research expressed their concern regarding possible adverse reactions and the medical staff was reported as an important/very important source of information [25,26,27,31,32]. Greenberg et al. also reported doctors and public health authorities as the most reliable sources of information about vaccination [34]. When asked if they trusted the information provided by medical personnel about vaccination, 85% of participants in another research answered yes [35]. The most credible sources of information in the context of a national information campaign, considered otherwise opportune/necessary by the majority of respondents in the study conducted by Voidazan et al., are the Ministry of Health (36.5%) and pediatricians (27.2%). Also, respondents from the urban environment and those with higher or high school education prefer the Internet and specialized literature, while the participants from rural environments turn to the family doctor and friends and those with secondary school education especially to the family doctor, according to the same research [36]. Paradoxically, another study reports doctors as the main source of contrary vaccination information (41%), followed by the Internet/TV (32%) [37]. Another study highlighted generally positive attitudes regarding vaccination, with most participants considering vaccines important for their own child’s health, but also for the protection of those around them [38].

The relationship between vaccine hesitancy and vaccination intention has been demonstrated in other studies. Also, the belief that the recommended schedules contain too many vaccines at too young an age was frequently associated with hesitant behavior and intention to vaccinate [25,39,40,41,42].

A study carried out in Italy, in the main commercial center of Palermo, through the application of a questionnaire followed by individual counseling on vaccination, highlighted that the specific intervention can positively influence perceptions and attitudes related to vaccination [43]. 

As we can see, the specialized literature highlights the fact that sociodemographic variables/characteristics influence and have different effects on perceptions and attitudes regarding vaccination, the results of the studies being different depending on the period of implementation, location and the population addressed. The results obtained in the present study allow the development and implementation of interventions targeted at the particularities of hesitant behavior. 

Healthcare professionals play a crucial role in advocating and disseminating positive messages about vaccines and vaccination; furthermore, as outlined by the specialized literature, medical advice can have an important impact on parent’s perception and intention related to vaccination. 

To ensure effective communication with the parent, medical professionals must have confidence in the safety, effectiveness and the importance of vaccination. Similarly to other research, our study has revealed that vaccine hesitancy can also affect healthcare professionals. In order to effectively address any concern, shortcomings must be identified, and educational resources must be provided to them [44,45,46]. 

ECDC developed a communication guide addressed to healthcare workers, health mediators and other professionals involved in vaccination services, as well as medical students. This guide was translated into Romanian and made available to all medical professionals. Additionally, the Bihor County Public Health Directorate holds periodic meetings with local general practitioners to disseminate information regarding vaccines and immunizations and to address any issues or concerns that arise in the region [47]. 

In Romania, there is a need for multidirectional strategies, in the sense of information campaigns regarding vaccines and vaccination, in which we must engage doctors directly involved in the vaccination process (neonatologists, pediatricians, family doctors), as well as local public health authorities (through specialists in epidemiology, public health, communication), local authorities (through social workers, community nurses and health mediators (especially where we are dealing with disadvantaged populations with very low vaccination rates and there is a need to mobilize them)) and representatives of other bodies and institutions that support and promote vaccination, but also community leaders, public figures, parents who want to get involved in transmitting positive messages about vaccines and vaccination, teachers, doctors and school assistants when we speak about HPV vaccination [48]. 

## 5. Conclusions

Identifying vaccine hesitancy in postpartum mothers allows tracking key particularities that can lead to optimizing interventions designed to respond to community members’ concerns, in order to increase vaccine coverage rates to levels that provide protection and prevent the occurrence of epidemics with potentially fatal consequences. Without trying to have similar samples in identifying the particularities of unhesitant/hesitant behavior, since the delimitation between the two groups was done at the time of interpreting the results, mothers with hesitant behavior represent almost half of the women participating in our study. The behavior of this group is influenced by the low level of knowledge and information about vaccination, concerns about adverse effects and a high perception of the risks related to vaccination, while the perception of the risks associated with the disease is low. The study aims to represent the starting point for the organization and running of information campaigns regarding vaccination at the level of Bihor County, especially in areas with low vaccination coverage, where this behavior is identified.

## Figures and Tables

**Table 1:** Exploratory Factor Analysis results.

	S2	S3	S1	S5	S4	S6
Sum of squared loadings	4.67	4.46	3.77	3.60	3.11	3.10
Proportion of variance	0.16	0.15	0.13	0.12	0.10	0.10
Cumulative proportion of variance	0.16	0.30	0.43	0.55	0.65	0.76

Note: S1 to S6 represent the subscales (factors) analyzed in the questionnaire.

**Table 2:** Demographics and general characteristics of the study GNV and GPV samples.

Characteristic	Category	Total Sample	GNV		GPV	
			Number	%	Number	%
		N = 404	191	47.28	213	52.72
Average age			29.43		28.99	
Environment	Rural	191	81	42.41	110	57.59
	Urban	213	110	51.64	103	48.36
Studies	General school	38	11	28.95	27	71.05
	High school/vocational school	141	68	48.23	73	51.77
	Postsecondary school	29	16	55.l7	13	44.83
	University	194	96	49.48	98	50.51
	No studies	2	-	-	2	100
Ethnicity	Romanian	353	170	48.16	183	51.84
	Hungarian	31	16	51.61	15	48.39
	Rome	19	4	21.05	15	78.95
	Other	1	1	100	-	-
Marital status	Married	348	174	50	174	50
	Single	48	14	29.17	34	70.83
	Divorced	8	3	37.5	5	62.5
Occupation	Medical staff/pharmacist	34	21	61.76	13	38.23
	Housewife/no occupation	104	43	41.35	61	58.65
	Other occupations	266	127	47.74	139	52.25
Number of children	First child/primiparous	208	112	53.85	96	46.15
	Multiparous	196	79	40.31	117	59.69

GNV—group non vaccine, GPV—group pro vaccine, N—number.

**Table 3:** Distribution of GNV and GPV responses to subscale 1—Knowledge about vaccination, general perceptions and perceptions of disease severity. Principal Component Analysis (PCA).

Item	Answers	GNV (191)		GPV (213)		GNV		GPV	
						PCA—Component			
		N	%	N	%	1	2	1	2
*S1.1. Vaccines prevent serious, potentially fatal diseases. Have you heard/read about this before completing this survey?*	Yes	151	79.06	183	85.91	0.545	−0.441	0.665	−0.339
	No	25	13.09	24	11.27				
	I don’t know/I’m not sure	15	7.85	6	2.82				
*S1.2. Vaccines prevent serious, potentially fatal diseases. Do you think this statement is:*	True	139	72.77	190	89.20	0.787	−0.030	0.733	−0.179
	False	5	2.62	4	1.88				
	I have no opinion	47	24.61	19	8.92				
*S1.3. Vaccines prevent serious, potentially fatal diseases. Does this statement influence your decision to vaccinate your child?*	Yes	109	57.07	142	66.67	0.709	0.042	0.620	−0.316
	No	57	29.84	64	30.04				
	I don’t know/I’m not sure	25	13.09	7	3.29				
*S1.4. How familiar are you with/know your child’s current recommended vaccination schedule?*	I didn’t know there was a vaccination schedule	31	16.23	20	9.39	−0.275	0.672	−0.386	−0.318
	I don’t know what the vaccines are in the calendar	24	12.56	29	13.61				
	I heard about some of the types of vaccine	95	49.74	96	45.07				
	I know/I have heard about all the vaccines in the schedule	41	21.46	67	31.45				
	I am not interested in this	-	-	1	0.47				
*S1.5. Do you think vaccines can protect your child against serious diseases?*	Yes	158	82.72	204	95.77	0.655	0.231	0.433	0.581
	No	8	4.19	1	0.47				
	I do not know	25	13.09	8	3.75				
*S1.6. Do you think vaccination of children should be compulsory?*	Yes	97	50.78	187	87.79	0.474	0.566	0.227	0.784
	No	48	25.13	6	2.82				
	I don’t know/I’m not sure	46	24.08	20	9.39				
Initial Eigenvalues						2.148	1.023	1.752	29.206
% of Variance						35.799	17.043	29.206	21.664

GNV—group non vaccine, GPV—group pro vaccine.

**Table 4:** Distribution of GNV and GPV responses to subscale 2—Perception of the importance, effectiveness, safety and benefits of vaccination. Principal Component Analysis (PCA).

Item	Answers	GNV (191)		GPV (213)		GNV	GPV
						PCA-Component	
		N	%	N	%	1	1
*S2.1. How important is it to you that this child receives all the vaccines in the national schedule?*	Very important	75	39.27	151	70.89	0.814	0.601
	Important	75	39.27	60	28.17		
	I do not know	30	15.70	2	0.94		
	Not very important	8	4.19	-	-		
	Not important at all	3	1.57	-	-		
*S2.2. Vaccines are very important to my child’s health*	I totally agree	59	30.89	139	65.26	0.836	0.784
	I agree	101	52.88	69	32.39		
	I do not know	20	10.47	5	2.35		
	I disagree	7	3.66	-	-		
	I don’t agree at all	4	2.09	-	-		
*S2.3. Vaccines given to children are effective in preventing disease*	I totally agree	51	26.70	124	58.21	0.870	0.814
	I agree	104	54.45	78	36.62		
	I do not know	30	15.71	11	5.16		
	I disagree	5	2.62	-	-		
	I don’t agree at all	1	0.52	-	-		
*S2.4. Vaccines given to children are safe*	I totally agree	21	10.99	60	28.17	0.804	0.734
	I agree	75	39.27	117	54.93		
	I do not know	79	41.36	36	16.90		
	I disagree	9	4.71	-	-		
	I don’t agree at all	7	3.66	-	-		
*S2.5. It is very important to vaccinate my child also for the health of the other members of the community we are part of*	I totally agree	26	13.61	89	41.78	0.792	0.732
	I agree	108	56.54	110	51.64		
	I do not know	44	23.04	12	5.63		
	I disagree	10	5.23	2	0.94		
	I don’t agree at all	3	1.57	-	-		
*S2.6. All vaccines given to my child/community through the vaccination program are beneficial*	I totally agree	22	11.52	87	40.84	0.876	0.833
	I agree	101	52.88	105	49.29		
	I do not know	55	28.79	21	9.86		
	I disagree	10	5.23	-	-		
	I don’t agree at all	3	1.57	-	-		
*S2.7. Vaccination is a very good way to protect my child*	I totally agree	35	18.32	96	45.07	0.882	0.766
	I agree	118	61.78	111	52.11		
	I do not know	28	14.66	5	2.35		
	I disagree	7	3.66	1	0.47		
	I don’t agree at all	3	1.57	-	-		
Initial Eigenvalues						4.937	3.994
% of Variance						70.524	57.064

GNV—group non vaccine, GPV—group pro vaccine.

**Table 5:** Distribution of GNV and GPV responses to subscale 3—Worries, concerns, perception of vaccination risk and disease risk. Principal Component Analysis (PCA).

Item	Answers	GNV (191)		GPV (213)		GNV		GPV
						PCA-Component		
		N	%	N	%	1	2	1
*S3.1. The new vaccines are not as safe and carry more risks than the old ones*	I totally agree	12	6.28	9	4.22	0.583	0.403	0.661
	I agree	34	17.80	21	9.86			
	I do not know	129	67.54	133	62.44			
	I disagree	14	7.33	44	20.66			
	I don’t agree at all	2	1.05	6	2.82			
*S3.2. I am concerned about side effects*	I totally agree	62	32.46	50	23.47	0.498	0.671	0.528
	I agree	94	49.21	108	50.70			
	I do not know	28	14.66	28	13.14			
	I disagree	7	3.66	24	11.27			
	I don’t agree at all	-	-	3	1.41			
*S3.3. My child does not need to be vaccinated against diseases that are currently rare*	I totally agree	19	9.95	10	4.70	0.626	0.061	0.665
	I agree	50	26.18	38	17.84			
	I do not know	84	43.98	75	35.21			
	I disagree	33	17.28	73	34.27			
	I don’t agree at all	5	2.61	17	7.98			
*S3.4. Children receive more vaccines than they should*	I totally agree	12	6.28	2	0.94	0.780	−0.258	0.727
	I agree	24	12.56	15	7.04			
	I do not know	110	57.59	102	47.89			
	I disagree	42	21.99	78	36.62			
	I don’t agree at all	3	1.57	16	7.51			
*S3.5. It would be better for my child to develop defenses through illness rather than vaccination*	I totally agree	4	2.09	3	1.41	0.587	−0.599	0.597
	I agree	20	10.47	17	7.98			
	I do not know	59	30.89	29	13.61			
	I disagree	91	47.64	117	54.93			
	I don’t agree at all	17	8.90	47	22.06			
*S3.6. It would be better for my child to receive fewer vaccines at the same time*	I totally agree	18	9.42	6	2.82	0.715	−0.077	0.627
	I agree	47	24.61	36	16.90			
	I do not know	87	45.55	95	44.60			
	I disagree	38	19.89	65	30.52			
	I don’t agree at all	1	0.52	11	5.16			
Initial Eigenvalues						2.445	1.049	2.437
% of Variance						40.747	17.483	40.615

GNV—group non vaccine, GPV—group pro vaccine.

**Table 6:** Distribution of GNV and GPV responses to subscale 4—Vaccination intentions. Principal Component Analysis (PCA).

Item	Answers	GNV (191)		GPV (213)		GNV		GPV
						PCA—Component		
		N	%	N	%	1	2	1
*S4.1. Which of the following* *statements best describes your plans for vaccinating your child?*	I intend to vaccinate my child with all the vaccines recommended by the doctor	117	61.26	213	100	0.703	0.037	0.123
	I plan to vaccinate my child with some vaccines, but not all	48	25.13	-	-			
	I do not plan to vaccinate my child	3	1.57	-	-			
	I haven’t decided yet/I don’t know	23	12.04	-	-			
*S4.2. I plan to vaccinate my child with the pneumococcal vaccine, a new vaccine introduced in the vaccination schedule, to protect against pneumococcal pneumonia*	Yes	83	43.45	165	77.46	0.730	0.049	0.735
	No	11	5.76	5	2.35			
	I don’t know/I’m not sure	97	50.78	43	20.18			
*S4.3. Would you like to vaccinate your child with other types of vaccine, which are not included in the national program?*	Yes	12	6.28	41	19.25	0.272	0.901	0.735
	No	80	41.88	74	34.74			
	I don’t know/I’m not sure	99	51.83	98	46.01			
*S4.4. Overall, how hesitant do you consider yourself to be about vaccinating your child*	I don’t hesitate at all	13	6.81	213	100	0.658	−0.467	0.163
	Not very hesitant	102	53.40	-	-			
	I’m not sure	48	25.13	-	-			
	Somewhat hesitant	24	12.56	-	-			
	Very hesitant	4	2.09	-	-			
Initial Eigenvalues						1.535	1.033	1.081
% of Variance						38.379	25.836	54.072

GNV—group non vaccine, GPV—group pro vaccine.

**Table 7:** Distribution of GNV and GPV responses to subscale 5—Trust in medical professionals. Principal Component Analysis (PCA).

Item	Answers	GNV (191)		GPV (213)		GNV	GPV
						PCA—Component	
		N	%	N	%	1	1
*S5.1. I trust the information I receive about the vaccines in the program*	I totally agree	10	5.23	57	26.76	0.802	0.735
	I agree	112	58.64	139	65.26		
	I do not know	52	27.22	15	7.04		
	I disagree	14	7.33	2	0.94		
	I don’t agree at all	3	1.57	-	-		
*S5.2. I generally do what my doctor recommends when it comes to vaccinating my children*	I totally agree	36	18.85	98	46.01	0.882	0.858
	I agree	134	70.16	110	51.64		
	I do not know	11	5.76	5	2.35		
	I disagree	7	3.66	-	-		
	I don’t agree at all	3	1.57	-	-		
*S5.3. I am open to discussing any issues or concerns about vaccines with my child’s doctor*	I totally agree	83	43.45	122	57.28	0.752	0.793
	I agree	98	51.31	89	41.78		
	I do not know	6	3.14	2	0.94		
	I disagree	2	1.05	-	-		
	I don’t agree at all	2	1.05	-	-		
Initial Eigenvalues						1.986	1.906
% of Variance						66.209	63.527

GNV—group non vaccine, GPV—group pro vaccine.

**Table 8:** Distribution of GNV and GPV responses to subscale 6—Sources of information. Principal Component Analysis (PCA).

Item	Answers	GNV (191)		GPV (213)		GNV		GPV	
						PCA—Component			
		N	%	N	%	1	2	1	2
*S6.1. Have you ever searched for information about vaccines and vaccination?*	Yes	154	80.63	168	78.87	0.597	0.170	0.650	0.037
	No	32	16.75	41	19.25				
	I don’t know/I don’t remember	5	2.62	4	1.88				
*S6.2. Where did you look/who did you talk to?*	GP	43	22.51	72	33.80	0.778	−0.001	0.719	0.323
	Pediatrician	4	2.09	7	3.29				
	Neonatologist	4	2.09	3	1.41				
	Other medical staff (nurse)	-	-	-	-				
	Pharmacist	2	1.05	1	0.47				
	Family members/friends	8	4.19	5	2.35				
	Internet	14	7.33	12	5.63				
	Medical book/journal	3	1.57	2	0.94				
	Another source	1	0.52	-	-				
	Multiple sources	79	41.36	70	32.86				
	I didn’t search	33	17.28	41	19.25				
*S6.3. Where do you usually look for information about vaccines?*	Google	73	38.22	76	35.68	0.515	0.065	0.400	0.492
	Medical websites	22	11.52	19	8.92				
	Forums for parents/pregnant women	9	4.71	16	7.51				
	Blogs for parents	3	1.57	4	1.88				
	Social networks	4	2.09	4	1.88				
	Applications	-	-	1	0.47				
	TV/Radio	3	1.57	-	-				
	I’m not looking for information	30	15.71	46	21.60				
	Multiple sources	47	24.61	47	22.06				
*S6.4. Did certain information you heard/seen make you change your mind about the decision to vaccinate your child?*	Yes	18	9.42	11	5.16	−0.138	0.807	−0.463	0.459
	No	173	90.58	202	94.84				
*S6.5. If a public figure, especially one you look up to, advocated against vaccination, would that influence your decision to vaccinate your child?*	Yes	14	7.33	3	1.41	−0.195	0.484	−0.411	0.730
	No	177	92.67	210	98.59				
*S6.6. Do you think you have enough information or sources of information available to make an informed decision about vaccinating your child?*	Yes	117	61.25	180	84.51	0.437	0.006	−0.073	−0.054
	No	74	38.74	33	15.49				
*S6.7. What information would you like to be made available to you?*	What is the risk of making the disease preventable by vaccination	27	14.14	33	15.49	0.364	0.359	0.378	0.393
	How serious is the vaccine preventable disease	10	5.23	11	5.16				
	The risk of adverse reactions after vaccination	54	28.27	52	24.41				
	Information on the vaccination schedule	3	1.57	11	5.16				
	Multiple answers	92	48.17	105	49.30				
	None	5	2.62	1	0.47				
*S6.8. What do you think is the most reliable source of vaccination information?*	The doctor	182	95.29	208	97.65	0.096	−0.645	0.271	−0.342
	The nurse	1	0.52	1	0.47				
	The Internet	5	2.62	4	1.88				
	TV/Radio	1	0.52	-	-				
	Another source	2	1.05	-	-				
Initial Eigenvalues						1.616	1.462	1.704	1.367
% of Variance						20.201	18.281	21.302	17.089

GNV—group non vaccine, GPV—group pro vaccine.

## Data Availability

The data presented in this study are available on request from the first author.
